# Morphogenesis along the animal-vegetal axis: fates of primary quartet micromere daughters in the gastropod *Crepidula fornicata*

**DOI:** 10.1186/s12862-017-1057-1

**Published:** 2017-09-15

**Authors:** Deirdre C. Lyons, Kimberly J. Perry, Jonathan Q. Henry

**Affiliations:** 1 Scripps Institution of Oceanography, University of California, San Diego, 9500 Gilman Drive, La Jolla, San Diego, CA 92093 USA; 2 Department of Cell & Developmental Biology, University of Illinois, 601 S. Goodwin Ave, Urbana, IL 61801 USA

**Keywords:** Axial elongation, Gastropoda, Lophotrochozoa, Morphogenesis, Mollusca, Prototroch, Spiralia, Trochoblasts

## Abstract

**Background:**

The Spiralia are a large, morphologically diverse group of protostomes (e.g. molluscs, annelids, nemerteans) that share a homologous mode of early development called spiral cleavage. One of the most highly-conserved features of spiralian development is the contribution of the primary quartet cells, 1a-1d, to the anterior region of the embryo (including the brain, eyes, and the anterior ciliary band, called the prototroch). Yet, very few studies have analyzed the ultimate fates of primary quartet sub-lineages, or examined the morphogenetic events that take place in the anterior region of the embryo.

**Results:**

This study focuses on the caenogastropod slipper snail, *Crepidula fornicata*, a model for molluscan developmental biology. Through direct lineage tracing of primary quartet daughter cells, and examination of these cells during gastrulation and organogenesis stages, we uncovered behaviors never described before in a spiralian. For the first time, we show that the 1a^2^-1d^2^ cells do not contribute to the prototroch (as they do in other species) and are ultimately lost before hatching. During gastrulation and anterior-posterior axial elongation stages, these cells cleavage-arrest and spread dramatically, contributing to a thin provisional epidermis on the dorsal side of the embryo. This spreading is coupled with the displacement of the animal pole, and other pretrochal cells, closer to the ventrally-positioned mouth, and the vegetal pole.

**Conclusions:**

This is the first study to document the behavior and fate of primary quartet sub-lineages among molluscs. We speculate that the function of 1a^2^-1d^2^ cells (in addition to two cells derived from 1d^12^, and the 2b lineage) is to serve as a provisional epithelium that allows for anterior displacement of the other progeny of the primary quartet towards the anterior-ventral side of the embryo. These data support a new and novel mechanism for axial bending, distinct from canonical models in which axial bending is suggested to be driven primarily by differential proliferation of posterior dorsal cells. These data suggest also that examining sub-lineages in other spiralians will reveal greater variation than previously assumed.

## Background

The body-plans of most bilaterians are patterned along three main axes [1, 2]. Generally, the anterior-posterior (A-P) axis is the longest axis, with a concentration of sensory organs associated with an anterior brain, and a posterior hindgut, terminating in an anus [3]. Orthogonal to the A-P axis is the dorsal-ventral (D-V) axis; typically the central nervous system is positioned dorsally in the deuterostome branch of bilaterians, and ventrally in the protostome branch. In both protostomes and deuterostomes, the mouth is situated in the anterior region on the ventral side [4]. The third, orthogonal, left-right (L-R) axis exhibits varying degrees of asymmetry, depending on the species. These axes arise during development and are not typically pre-patterned in the egg. The egg often has a distinct set of patterning cues that initially operate along the primary, or animal-vegetal (A-V) axis. As development proceeds, the A-V axis is transformed, and additional axes are added; ultimately the definitive A-P, D-V, and L-R axes are established by the deployment of zygotic gene regulatory networks, and the movement and rearrangement of cells through morphogenesis. These morphogenetic events are well-understood at the molecular and cellular levels in several deuterostomes (e.g. sea urchins, ascidians, vertebrates; [5–7], and ecdysozoans (e.g. arthropods, nematodes, [8, 9]). But few studies have documented even the basic morphogenetic events involved in this transformation in the third major branch of bilaterians, the Spiralia/Lophotrochozoa [10–14].

The Spiralia/Lophotrochozoa are a large branch of protostomes, comprising ~14 “phyla” with diverse body-plans (e.g., molluscs, annelids, brachiopods, phoronids, and rotifers and other minor taxa [15–18]). Despite looking very different as adults, many of these groups (e.g. molluscs, annelids, nemerteans, platyhelminths) share a highly stereotyped, homologous suite of characteristics during early development, including spiral cleavage [15, 16]. Embryos exhibiting spiral cleavage are initially patterned along the primary, animal-vegetal axis [19, 20]. The animal pole gives rise to cell lineages that will become neural tissues in the head, while cell lineages born from more vegetal territories give rise to neurons in the trunk, and epidermal, mesodermal, and endodermal tissues. Patterning along the dorsal-ventral axis is established by a dorsal organizer, which signals prior to gastrulation [21–23].

Gastrulation movements radically transform the embryo [24]. During gastrulation in bilaterians, mesoderm and endoderm are internalized through the blastopore, which forms at the vegetal pole [4]. In deuterostomes, gastrulation occurs at the vegetal pole, and the blastopore becomes the opening to the posterior anus; the mouth forms separately within the anterior/ventral territory. In contrast, among the Spiralia, the relationship between the blastopore, the mouth, and the anus is still debated [10, 13, 25–28]. This is because fewer studies have been performed using spiralians to follow cells around the blastopore to discern their ultimate contribution to the mouth or anus using modern intra-cellular lineage tracing [29, 30]. Furthermore, the blastopore forms at the vegetal pole, but the mouth, which it typically gives rise to, is ultimately situated in the anterior, ventral territory in spiralians [4]. During these events the animal-vegetal axis is bent, and the original animal-vegetal axis of the egg cannot be directly correlated with the anterior-posterior axis. Thus, which cells surround the blastopore, what they ultimately contribute to, and how the blastopore becomes displaced from the vegetal pole towards the ventral/anterior region, is largely unknown.

Recently, we used intra-cellular lineage tracing and live imaging in the slipper snail *C. fornicata* to examine how the blastopore narrows at the vegetal pole [29]. We discovered that in this species it narrows in a posterior-to-anterior direction through convergence and extension that involves intercalation of cells across the midline, along the posterior edge of the blastopore lip. We also documented the further maturation and displacement of the blastopore/nascent mouth towards the anterior-ventral region as the embryo is transformed from a sphere of blastomeres to an elongated, organogenesis stage.

**Fig. 1:**
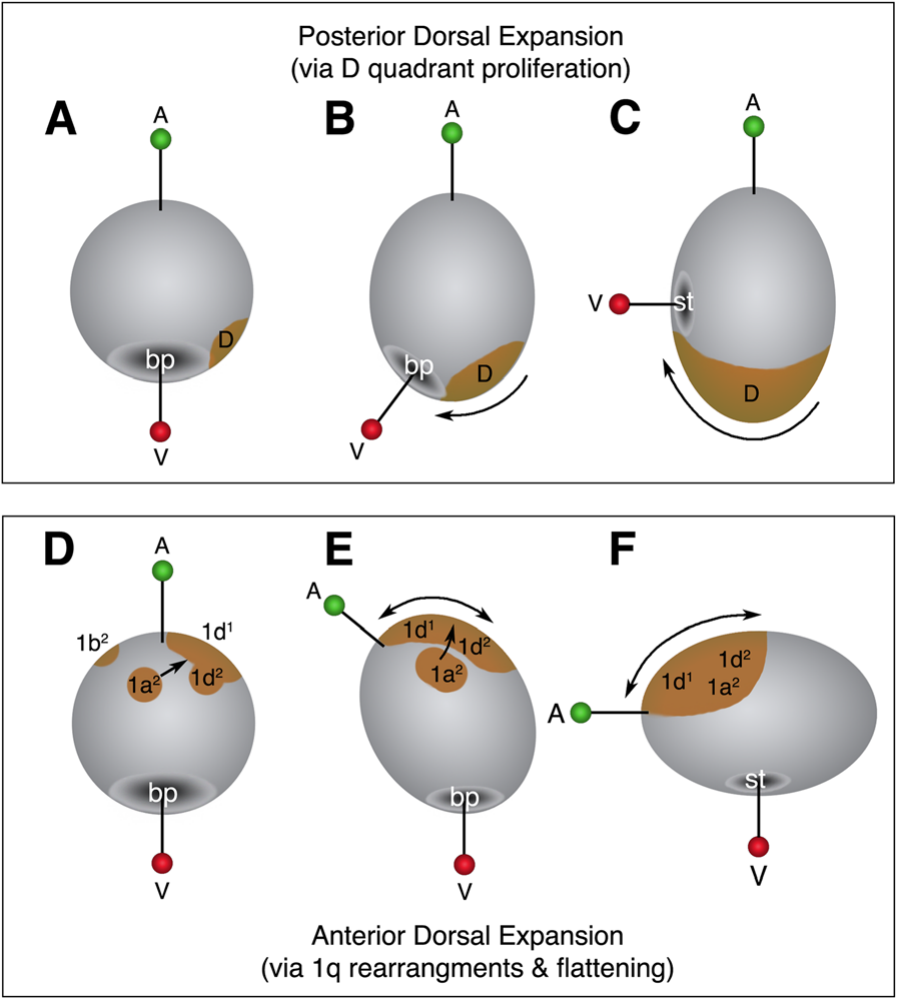
Proposed models describing morphogenetic events that contribute to the bending of the animal-vegetal axis of the spiralian embryo. These events reposition the mouth to the future ventral side of the embryo and closer the animal (future anterior) pole. **a**-**c** Earlier model suggesting that the vegetal pole and site of gastrulation (where the blastopore and mouth form) is displaced by differential proliferation of post-trochal D quadrant progeny (mainly 2d progeny) on the posterior dorsal side of the embryo [32]. **d**-**f** Model proposed here based on data from *C. fornicata* where axial bending is driven by ventral displacement of the animal pole through rearrangement and flattening of 1q^2^ (i.e., 1a^1^-1d^1^) and 1d^1^ (i.e., 1d^121^ and 1d^122^) progeny. While these two models are not mutually exclusive, the data reported here revealed the latter process in *C. fornicata*, and indicate that these cells are repositioned towards the dorsal side of the head to form an expansive, provisional epithelium, which is subsequently lost before hatching. Cells are labeled following the nomenclature of Conklin [40], and as described in the text. bp, blastopore; A, animal pole; st, stomodeum; V, vegetal pole

The mechanisms driving axial bending, however, and the concomitant anterior displacement of the blastopore is not well-understood among spiralians [10, 13]. Based on early studies of the pulmonate snail *Lymnaea stagnalis,* and others, the conventional explanation is that differential proliferation of cells on the post-trochal (posterior to the prototroch) dorsal side drives this process, pushing the blastopore and future mouth ventrally, closer to the pre-trochal animal pole region [31, 32] (see Fig. 1a-c). Early formation of the posterior-dorsal shell gland in molluscs has also been attributed to this axial displacement [33]. Some bending at the animal pole has been mentioned as playing a role in this process, but no specific cellular behaviors have been attributed to this process [34]. More recently, Maslakova et al. [11] showed that in the palaeonemertean *Carinoma tremaphoros,* expansion of cells in the dorsal pre-trochal (anterior to the prototroch) region is involved in pushing the dorsal prototroch band towards the posterior end of the larva. This unusual behavior was only obvious upon careful examination of development using confocal microscopy, and in light of cell lineage analysis [34]. Thus, it is likely that closer examination of additional spiralians will uncover novel morphogenetic behaviors.

We present one such example in this study, gleaned from cell lineage analysis and examination of cell behavior of the primary quartet micromeres (1a, 1b, 1c and 1d, also referred to more generally as 1q cells) in *C. fornicata*. This species has been developed as a model system for spiralian embryogenesis [35, 36], and many details about cell division patterns, cell behavior, and ultimate contribution to the veliger larva are already understood [29, 37–39]. However, the primary quartet lineages had not been previously studied in detail during the interval between their earliest division patterns and their ultimate contribution to the anterior regions of the veliger larva. By examining intermediate stages of development, we observed several features of their behavior that have not previously been described in this species, or any other spiralian. First, we found that only the animal daughter cells of each primary quartet blastomere, the 1q^1^ cells (1a^1^, 1b^1^, 1c^1^, 1d^1^), contribute to the mature prototroch; the other daughters, the 1q^2^ cells (1a^2^, 1b^2^, 1c^2^, 1d^2^), are ultimately shed from the embryo before hatching. Second, we document how the four 1q^2^ cells, plus two cells derived from the 1d^1^ lineage (1d^121^ and 1d^122^), become cell-cycle arrested and undergo a dramatic cell flattening event, collectively forming an expansive provisional epithelium that covers the anterior-dorsal portion of the embryo during axial bending and elongation. These data provide an alternative (though not mutually exclusive) model of how a spiralian embryo transforms from the rounded cleavage-stages to the elongated larva (Fig. 1d-f). We discuss the significance of these morphogenetic behaviors in a comparative context.

## Results

### Overview of *C. fornicata* development

**Fig. 2:**
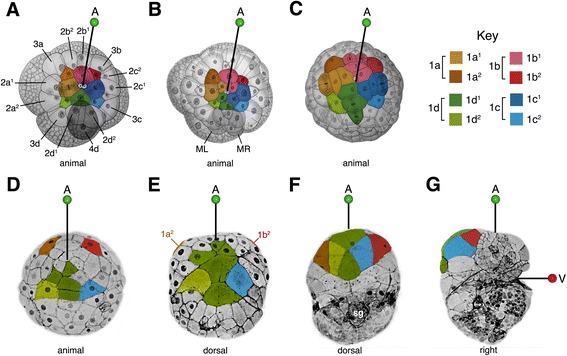
Schematic diagram highlighting cells/clones derived from the first quartet micromeres 1a^1^-1d^1^ and 1a^2^-1d^2^. These cells/clones are color coded as indicated in the key. **a**-**c** Animal pole views with the **d** quadrant located towards the *bottom* of the figure. The other micromere progeny are labeled in (**a**) for reference. The central small round polar bodies (*gray color*) are located at the animal pole. Note that the 1q^2^ cells do not undergo subsequent divisions and initially present a very small exposed area on the surface of the embryos, but eventually they spread out to occupy a greater exposed surface area (also see Fig. 3). **d**-**g** Diagrams showing later stages and differential movement of the 1q^2^ micromeres to form the provisional epithelium, with progeny of 1d^1^, on the dorsal side of the head. Color has been removed from the 1q^1^ clones for better clarity. These rearrangements help accommodate the relative displacement of the animal pole towards the vegetal/ventral pole during development. The posterior pole is located towards the *bottom* of the figure for embryos shown in (**d**-**g**). **d** Dorsal view during late cleavage in an embryo undergoing compaction. **e** Dorsal view of an embryo beginning elongation. **f** Dorsal view of an older embryo that is beginning organogenesis. The circular, condensed shell gland (sg) has begun to form in this embryo. **g** Right lateral view of an embryo that is somewhat older than that in (**f**). Note the animal pole ends up as the anterior pole of the embryo/larva while the vegetal pole (the site of gastrulation, blastopore, and mouth) ends up on the ventral surface. The originally straight animal-vegetal axis becomes bent, and the animal pole becomes located at 90 degrees relative to the vegetal/ventral pole. For each embryo, the animal/anterior pole (A) is indicated with a green-headed pin. The vegetal/ventral pole (V) is indicated with a red-headed pin in g. Embryos in (a-c) follow illustrations of Conklin [40]. Embryos in (**d**-**g**) are from confocal images where the cell outlines are visualized by expression of GFP-tagged UTPH (from [29])

As in other spiralians, *C. fornicata’s* first two cell divisions of the zygote, which are essentially equal, give rise to four blastomeres, termed A, B, C and D [40]. Subsequently, each of these cells will form successive tiers of smaller animal pole daughter cells called micromeres. The first tier, or primary quartet (1q) consists of the 1a-1d micromeres, while the corresponding larger vegetal macromeres are termed 1A -1D. These four macromeres in turn form the second, third, and finally the fourth quartet tier of micromeres (Fig. 2a). These micromeres undergo subsequent divisions (Fig. 2a-c). According to the nomenclature used by Conklin [40], those progeny born closer to the animal pole receive a superscript 1 (e.g., 1a^1^) while those born closer to the vegetal pole receive a 2 (e.g., 1a^2^, Fig. 2a). In *C. fornicata* the first quartet micromeres begin to divide after the 12-cell stage, following the birth of the second quartet [i.e., 2q (2a-2d), to reach the 16-cell stage]. The third quartet is formed next (3q at the 20-cell stage) and then all four 2q cells divide (forming the 2q^1^ and 2q^2^ cells to reach the 24-cell stage). Following this, the 4d cell is born precociously (i.e., the 25-cell stage), on the dorsal side, at approximately 25 h after fertilization, as shown in Fig. 2a. The other fourth quartet micromeres 4a-4c are born much later, at approximately 48 h of development [29, 37].

**Fig. 3:**
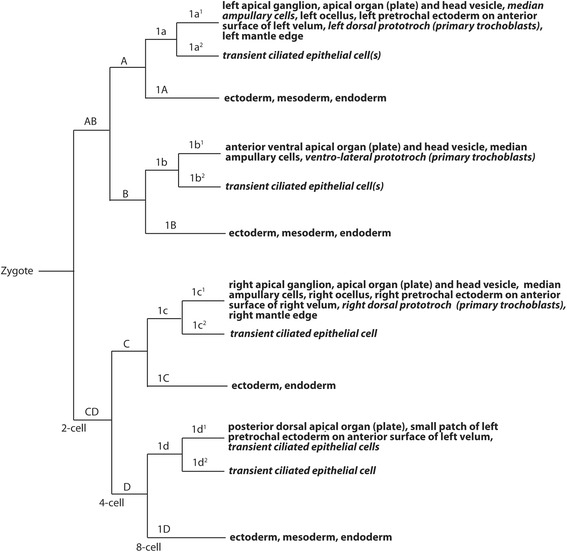
Cell lineage fate map for *C. fornicata*, summarizing the results presented here for the first quartet progeny (see also [37]). Nomenclature follows that of Conklin [40]. Fates shown in italics highlight information gleaned from the present study

The fate maps of sub-lineages within the 2q and 3q quartets, and within the 4d cell, have recently been examined in detail [29, 39]. In contrast, the fates of the sub-lineages of the 1q quartet are not known. Previous fate-mapping of the 1q cells at the veliger stage demonstrated that like other spiralians, the *C. fornicata* 1q cells give rise to the ectodermal structures of the head and the ciliated prototroch of the veliger [37, 38]. These analyses showed that the primary quartet micromeres give rise to several cell types including apical ganglia, apical organ, the ocelli, ampullary cells, ciliated cells of the prototroch, pretrochal epidermis of the head vesicle, and the anterior surface of the velar lobes (summarized in Fig. 3, with the new data presented below).

### Cleavage patterns and fates of the 1q^1^ and 1q^2^ progeny

**Fig. 4:**
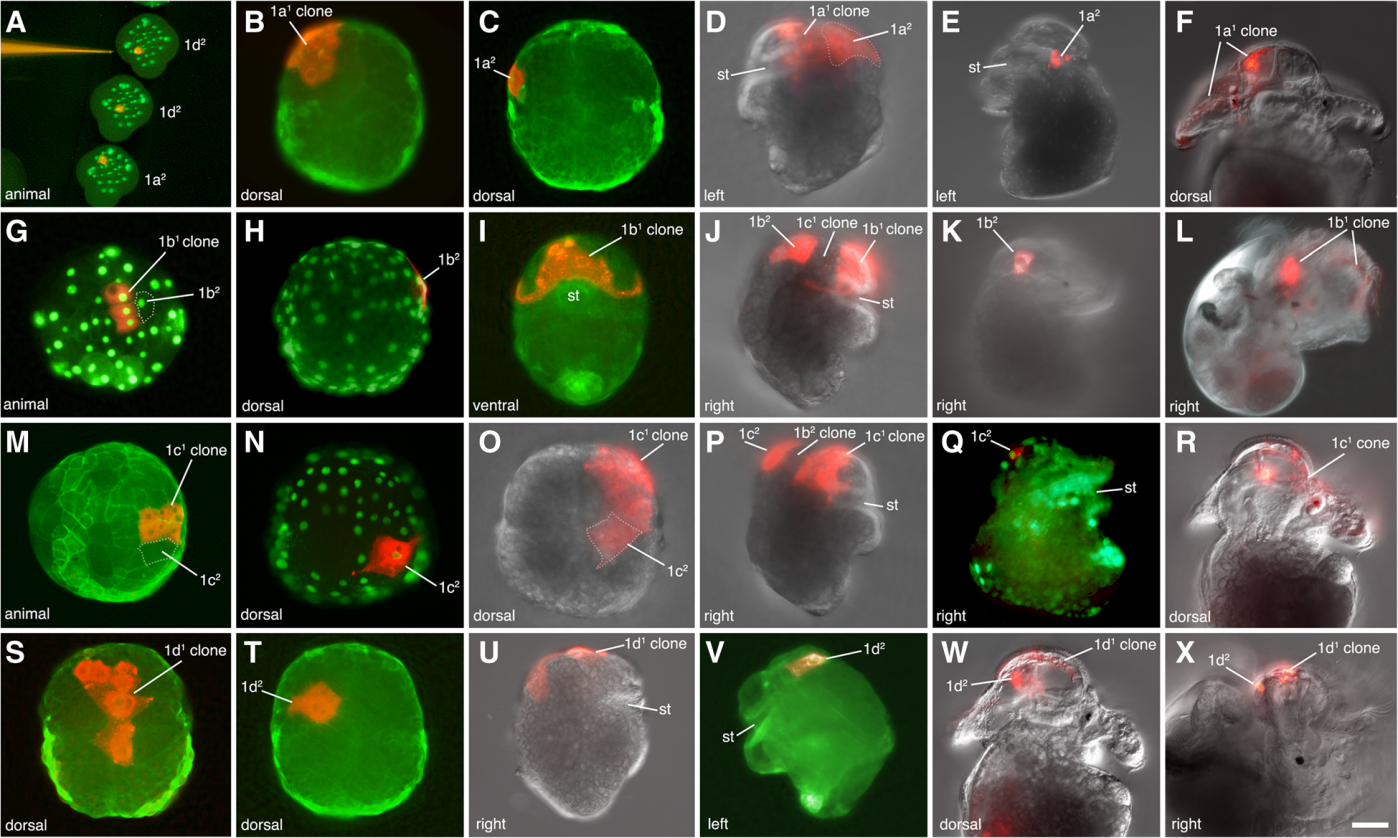
Live images showing examples of specific clones at various stages of development, as labeled. Individual cells were initially injected at the 16- to 28-cell stages. **a** Three examples of freshly injected 1q^2^ cells prior to their division (i.e., 1a^2^ and 1d^2^ at 28-cell stage, as labeled). Note the small apparent size of these micromere daughters. Nuclei were pre-labeled by expression of green GFP-histone H2B. *Red* fluorescent diI has been pressure microinjected into single cells using a fine glass needle, as shown. **b**-**f** 1a^1^ and 1a^2^ progeny, as labeled. **b**, **c** Dorsal-animal views of elongating embryos. **d**, **e** Left-lateral views of pre-veliger larvae undergoing organogenesis. **f** Anterior-dorsal view of an early veliger larvae. **g**-**l** 1b^1^ and 1b^2^ progeny, as labeled. **g** Animal view with the **d** quadrant located towards the *bottom* of the figure. **h** Dorsal-animal view. **i** Ventral view. **j**, **k** Right lateral views of pre-veliger larvae undergoing organogenesis. **l** Right lateral view of veliger larva. **m**-**r** 1c^1^ and 1c^2^ progeny, as labeled. **m** Animal pole view. **n** Dorsal-animal view. **o** Dorsal view. **p**-**q** Right-lateral views of pre-veliger larvae undergoing organogenesis. **r** Anterior-dorsal view of an early veliger larvae. **s**-**x** 1d^1^ and 1d^2^ progeny, as labeled. **s**-**t** Dorsal views of elongating embryos. **u**-**v** Right- and left-lateral views, respectively, of pre-veliger larvae undergoing organogenesis. **w**-**x** Anterior-dorsal and right-lateral views of early veliger larvae, respectively. To facilitate the injections embryos were pre-labeled by expression of either *green* GFP-histone H2B (as shown in **a**, **g**, **h**, **n**, **q**) or GFP-UTPH (as shown in **b**, **c**, **i**, **m**, **s**, **t**, **v**) protein. Unless noted otherwise, embryos are oriented with the posterior pole directed toward the *bottom* of the figure. Scale bar in X equals 50 μm

The 1a-1d micromeres are equal to one another in size at birth (8-cell stage at approximately 12 h of development) and are approximately 40 μm in diameter when viewed from the animal pole. By 15 h (at 23 °C), the 1a-1d cells divide asymmetrically, forming the larger 1q^1^ cells at the very animal pole, and the smaller 1q^2^ cells, located peripheral to them (Fig. 2a). The 1q^1^ and 1q^2^ cells are approximately 30 μm and 15 μm in diameter, respectively, when viewed from the animal pole. In comparison to the 1q^1^ cells, the 1q^2^ cells appear as tall narrow (columnar) cells during the early stages of development (Fig. 2a). Both the 1q^1^ and 1q^2^ cells are located at the surface of the embryo and are large enough to inject directly (Fig. 4a), thus their subsequent divisions and ultimate fate could be readily determined for this study.

Conklin [40] has described the early cleavage pattern of the 1q cells. Here we provide additional details related to the timing of these divisions and location of their progeny. At later stages (30–90 h) the embryos undergo compaction and appear very round. Therefore, obvious cellular landmarks are missing, so we note the absolute time of certain cell divisions, and correlate their divisions relative to those within the 4d lineage, whose cleavage patterns and division timing was established previously [39]. Immediately after the formation of the mesentoblast (4d at the 25-cell stage), the 1q^1^ cells divide to form 1q^11^ and 1q^12^ (achieving the 29-cell stage). Within two hours (after the 25-cell stage is reached, 27 h), the 4d cell divides to form bilaterally symmetrical teloblasts, ML and MR (at approximately 29 h, Fig. 2b, [39, 41], and unpublished data). After the formation of ML and MR, the next cells to divide are the 1q^12^ cells that give rise to 1q^121^ and 1q^122^, and by the time the 4a-4c micromeres are born (at approximately 47–48 h) there are three progeny derived from each of the 1q^1^ cells (1q^11^, 1q^121^ and 1q^122^), and the four 1q^2^ cells remain undivided (Fig. 2c). At that stage, there are six progeny of 4d [39, 42]. As epiboly begins (~ 48 h), the cleavage pattern of the 1d lineage becomes asynchronous and lags behind that of the 1a-1c lineages. The 1a^122^-1c^122^ cells divide to form 1a^1221^-1c^1221^ and 1a^1222^-1c^1222^, while the 1d^121^ and 1d^122^ cells remain undivided. Following these divisions, the 1a^121^, 1b^121^, 1c^121^ cells each divide, forming 1a^1211^-1c^1211^ and 1a^1212^-1c^1212^. This establishes the complement of 1q progeny at ~60 h (50–55% epiboly).

**Fig. 5:**
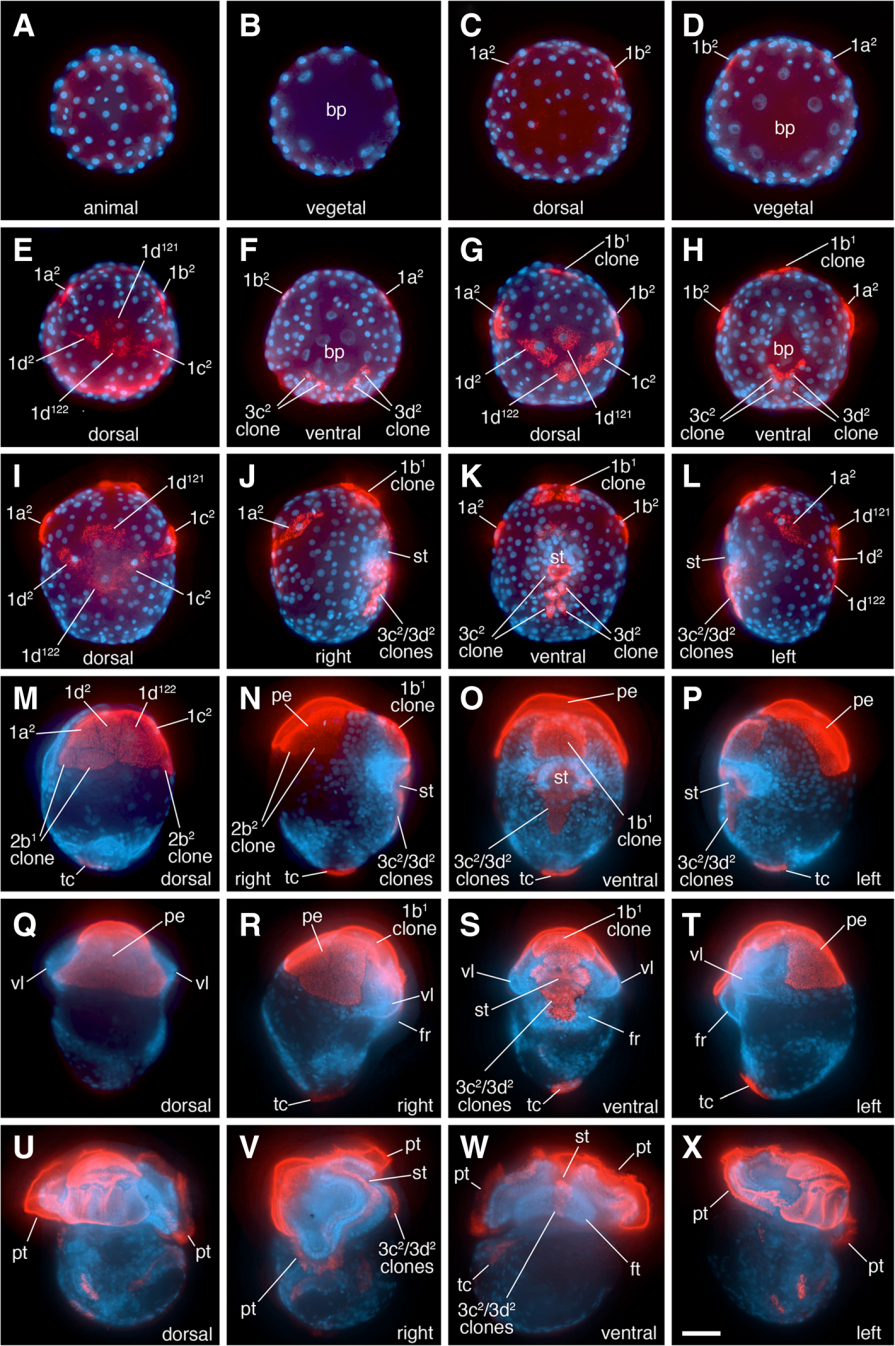
Anti-acetylated tubulin staining (*red fluorescence*) revealing ciliated cells in developing embryos. Embryos are oriented with the future posterior pole toward the *bottom* of the figure. Nuclei are labeled with DAPI (*blue fluorescence*). Specific cells/clones are as labeled. **a**, **b** Animal and vegetal views during early gastrulation, respectively. No ciliation is present at this stage. **c**, **d** Animal and vegetal views, respectively, during intermediate stages of gastrulation when ciliation is present in some 1q^2^ daughter cells, as labeled. **e**, **f** Animal-dorsal, and vegetal views later during gastrulation, respectively. Note group of four large ciliated cells on the dorsal surface in addition to 1a^2^ and 1b^2^. **g**, **h** Dorsal and ventral views, respectively, at an even later stage of gastrulation. Note even closer location of 1a^2^ and 1b^2^ relative to the four large ciliated cells on the dorsal surface (**g**). **I**-**L** Dorsal, right-lateral, ventral and left-lateral views of elongating embryo, respectively. Ciliated cells of the neurotroch (3c^2^ and 3d^2^ progeny) are seen within and posterior to the stomodeum [29]. The ciliated 1q^2^ cells along with 1d^121^ and 1d^122^ now occupy a large area on the dorsal-lateral surfaces of the future head. **m**-**p** Dorsal, right-lateral, ventral and left-lateral views, respectively, of a later stage when the embryo is undergoing elongation. Note large area occupied by ciliated cells on the dorsal surface of the head that contribute to the “provisional epithelium.” Additional cells have developed ciliation including progeny of 1b^1^, which will contribute to the anterior head field and prototroch, in addition to some progeny of 2b^1^ and 2b^2^. **q**-**t** Later stage of development when organ rudiments form. A large area is occupied by the provisional epithelium on the dorsal surface of the head. **u**-**x** Very early veliger larval stage. At this stage the ciliated prototroch cells have formed. There are ciliated cells in the head field and along the ventral midline in the foot (derived from 3c^2^ and 3d^2^). bp, blastopore; fr, foot rudiment; ft., foot; st, stomodeum; tc, terminal (“anal”) cells; vl, velar lobes; vr, velar rudiment. Scale bar in X equals 50 μm

**Fig. 6:**
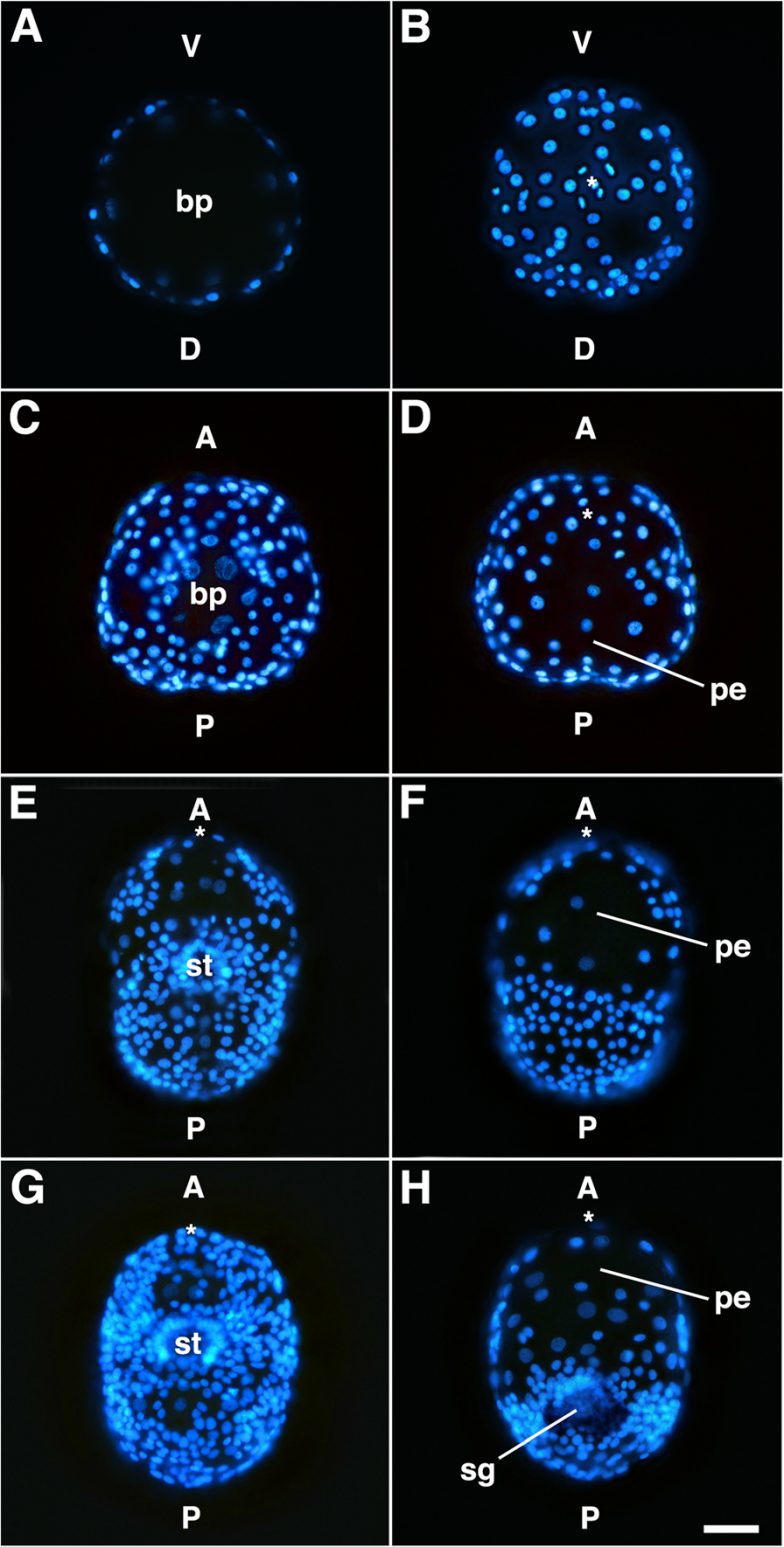
Embryos stained with DAPI to show the distribution of nuclei at four successive stages of development in *C. fornicata*. The formation of the expansive provisional epithelium, comprised of a small number of cells (nuclei) can be seen in (**d**, **f**, and **h**). **a**-**b** Corresponding fluorescence micrographs showing the vegetal (**a**) and animal (**b**) sides, respectively in a round stage embryo undergoing early stages of epiboly. The large opening of the blastopore is seen in (**a**), which is sparsely populated with cells, as the progeny of the animal micromeres have not yet made it to the vegetal side. Notice the fairly symmetrical pattern of the cells (nuclei) in the animal hemisphere in (**b**). **c**-**d** Corresponding fluorescence micrographs showing the vegetal (**d**) and animal (**d**) sides, respectively, of a flattened embryo undergoing later stages of epiboly. **c** The blastopore has closed considerably. **d** Notice that cells are somewhat more dispersed on the future dorsal side of the embryo as the provisional epithelium is beginning to form. **e**-**f** Corresponding fluorescence micrographs showing the ventral and dorsal sides, respectively, of an embryo undergoing elongation. The provisional epithelium is forming on the dorsal surface. **g**-**h** Corresponding fluorescence micrographs showing the ventral and dorsal sides, respectively, of an older embryo that has started organogenesis. **h** The provisional epithelium is present on the dorsal surface of the head, and the shell gland has begun to form in the post-trochal region. Additional nuclei appear to be present in the region of the provisional epithelium, as the deeper endodermal macromeres have undergone some divisions by this stage of development. a, anterior; bp, blastopore; d, dorsal; P, posterior; pe, provisional epithelium; sg, shell gland; st, stomodeum; V, ventral. Asterisk in **b**, **d**, **e**-**h** marks the location of the animal pole. Scale bar in h equals 50 μm

**Fig. 7:**
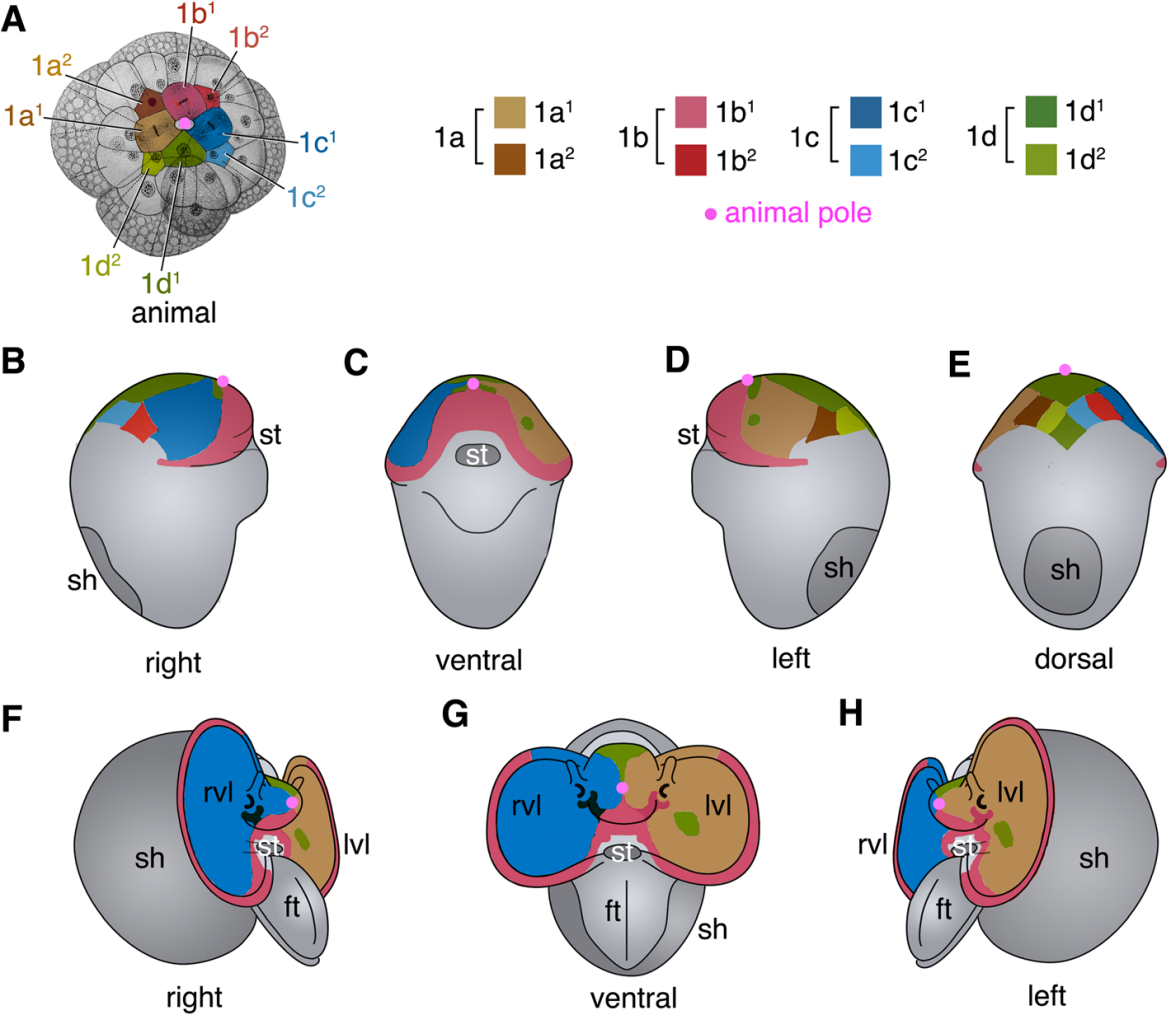
Summary diagram showing behavior of the 1q^2^ micromeres and 1q^1^ progeny at later stages of development. Individual cells and their clones are colored, as shown in (**a**). Orientations or views are indicated under each diagram. Embryos (**b**-**h**) are oriented with the future posterior pole directed toward the bottom of the figure. The animal pole is marked with a *pink dot* in each embryo. **a** Animal pole view of 25-cell stage and color key. **b**-**e** Four different views of an early organogenesis stage embryo showing contributions of the 1q progeny to the developing head. Note that the 1q^2^ cells now occupy a much smaller area on the dorsal surface of the head. **f**-**h** Three views of an advanced veliger larva showing contributions of the formation of the head, anterior surface of the velum and the prototroch by progeny of the 1q^1^ cells. By this stage the 1q^2^ cells have been lost from the embryo. ft., foot; lvl, left velar lobe; rvl, right velar lobe; sh, shell; st, stomodeum. Based on the result of this study, as well as those of Hejnol et al. [37]

The behavior of the 1q^1^ and 1q^2^ clones during successive stages of development is highlighted in Figs. 2 and 4. During gastrulation the embryo flattens along the animal-vegetal axis (Fig. 2c-e) and the 1d^2^, 1d^121^/1d^122^, and 1c^2^ occupy a central, dorsal position (Fig. 2e; Fig. 4n-p, s-t; Fig. 5e–i). These four cells remain undivided and flatten considerably to spread out over the dorsal surface of the head, while the other 1q^1^ cells continue to proliferate (Fig. 4b–s). The originally ventral 1a^2^ and 1b^2^ cells remain separated from the contiguous 1d^2^, 1d^121^, 1d^122^ and 1c^2^ cells until about 130 h (Figs. 2d-e; 4c, h; 5e, g, i-j, l). The 1a^2^ and 1b^2^ cells are eventually relocated laterally and dorsally to take residence next to the 1d^2^ and 1c^2^ cells, respectively (Fig. 2d-g; Fig. 5i-m). This group of six cells—1a^2^, 1b^2^, 1c^2^, 1d^2^, 1d^121^ and 1d^122^ forms an expansive plate of thin, ciliated cells on the dorsal anterior side of the embryo by 130–137 h (Figs. 2f; Fig. 5m–p). At the border of these large cells, one can see extensive cellular processes (filopodia and lamellipodia). Throughout this time and continuing through later stages of development, the 1q^2^ cells in all four quadrants generally do not undergo division, though on rare instances we have seen that 1a^2^ and 1b^2^ may divide only once (see also Conklin, [40]). This large expanse of cells is also obvious when one simply stains embryonic nuclei with Hoechst. The large area that forms on the dorsal side of the head can be readily seen as one sparsely populated with nuclei (Fig. 6a-h). During later organogenesis stages through veliger stages (Fig. 4e–v-x), the four 1q–derived large flat cells gradually shrink to become tiny cells located at the surface of the head. Eventually these cells are lost from the embryo well before hatching (summarized in Fig. 7b-h). In other spiralians, the 1q^2^ cells typically give rise to the primary trochoblast cells. Therefore, in *C. fornicata*, the 1q^1^ cells give rise to all primary quartet-derived neural and epidermal pre-trochal structures, as well as the primary ciliary band of the prototroch itself (Fig. 4f–w-x, see [37]). The fates of the 1q^1^ and 1q^2^ progeny are summarized in Fig. 3.

As the 1a^2^ and 1b^2^ cells flatten out and make their way laterally to the dorsal surface of the embryo, the ventral progeny of 2b^1^ and 2b^2^ extend two bands of cells laterally to encircle the embryo, meeting at the dorsal midline (described in [29]). Some progeny derived from 2b^1^ and 2b^2^ also join this expanded 1q^2^ epithelium (see Fig. 5m-n, and further descriptions provided below).

### Differentiation of 1q^1^ cells: Patterns of ciliation

Given the unexpected result that 1q^2^ cells do not contribute to the larval tissue post-hatching, we examined earlier ciliation patterns via acetylated tubulin staining (Fig. 5a-x). This antibody also marks ciliated cells within other lineages (i.e., from the 2b, 3c, and 3d lineages), which are clearly distinguishable from the 1q progeny, as examined in a previous study [29]. The first cells to become multi-ciliated are the 1a^2^ and 1b^2^ cells, at ~ 90 h of development (Fig. 5a-d). These are joined by the 1c^2^ and 1d^2^ cells, the 1d^121^ and 1d^122^ cells, and animal-ventral cells derived from the 1b^1^ lineage (Fig. 5e-h). Following these stages, ciliation appears in the progeny of 3c^2^ and 3d^2^ along the posterior edge of the blastopore during early stages of elongation, (117–120 h, Fig. 5i-l). By 120 to 170 h, six highly ciliated, flattened cells (1a^2^, 1b^2^, 1c^2^, 1d^2^, 1d^121^ and 1d^122^) make up an expansive provisional epithelium located mainly on the dorsal surface of the developing head (Fig. 5m-t). Ciliation also develops in a few lateral progeny of the 2b^1^ and 2b^2^ cells, which lie just posterior to the 1a^2^ and 1b^2^ cells (Fig. 5m-n) and those cells could be contributing to the expansion of this epithelial territory. Ciliation in the 1q^2^ cells could represent a vestige of their protrochal ancestry, even though they do not contribute to the definitive prototroch of the veliger larva in *C. fornicata*.

Ciliation does not develop in the genuine prototrochal progeny of 1a^1^, 1b^1^ and 1c^1^ until much later in development, when organogenesis has already started to take place (~228 h, Fig. 5u-x, note that 1d^1^ does not contribute to the prototroch, Fig. 3). Additional cells derived from the 2b lineage also become ciliated at these later stages of development, and these cells contribute to the metatroch, a secondary ciliated band that sets up a reverse current used to capture food particles [37, 38].

## Discussion

### Morphogenesis of embryonic axes

Classical explanations for how the blastopore and mouth are relocated from the vegetal pole towards the ventral, anterior territory (which contributes to the bending of the primary, animal-vegetal axis) typically invoke proliferation in the dorsal, posterior (post-trochal) territory; this process is thought to drive the vegetal blastopore/mouth ventrally, and anteriorly (Fig. 1a-c, [10, 31, 32, 43]). In contrast, as schematized in Fig. 1d-f, in this study we observed an expansion of tissue in the dorsal *anterior, pre-trochal* region. We note it is possible that both mechanisms work together to drive axial bending. In *C. fornicata*, we found that all four 1q^2^ micromeres, plus two cells from the 1d^1^ lineage (1d^121^ and 1d^122^) become cleavage-arrested, and flatten, to form an expansive, thin ciliated provisional epithelium on the dorsal surface of the head (Fig. 2; Fig. 4d, j, n, o, p, s-t; Fig. 5 m–t; Fig. 6b, d, f, h). Conklin [40] referred to these cells collectively as the “posterior cell plate,” but failed to recognize their true fate and significance. As these cells flatten, and expand to cover more surface area, we propose that they serve to allow for the displacement of the animal pole and the other pretrochal 1q progeny towards the ventral mouth, and the vegetal pole (Fig. 1d-f). It is unclear if the cells within this provisional epithelium serve to actively push these other pretrochal cells**,** or if they are passively pulled (stretched) by the adjacent cells. Preliminary observations suggest it could be the latter, as we have used a laser to burst these cells and the remaining cells retract, as if they are under some tension (data not shown). The provisional epithelium may also contribute to elongation of the embryo (lengthening along the dorsal surface of the embryo), though there are likely other factors that contribute to the process of anterio-posterior axial elongation.

Previously [29], we found that the progeny of the 2b micromere (derived from 2b^1^ and 2b^2^), which are initially located on the anterior ventral side of the embryo (just anterior to the blastopore and developing mouth), form two bilateral bands of cells that wrap progressively around the left and right sides, ultimately joining on the dorsal side below the provisional epithelium. It is possible that the rearrangements of these 2b derived cells is coupled with the dorsal displacement of the 1a^2^ and 1b^2^ cells, which were also originally located on the ventral and lateral sides of the embryo (Fig. 2d-e; 4c, h; 5c–l). Some large cells derived from 2b^1^ and 2b^2^ also become ciliated adjacent to the provisional epithelium (Fig. 5m-n). Eventually other cells must replace the provisional epithelium, as those 1q^2^ cells decrease in size and are eventually lost from the embryo. Those cells could be derived from the progeny of 2b^1^ and 2b^2^, though we have not yet followed these later events.

Ventral displacement of the blastopore and mouth and the bending of the animal-vegetal axis, which reduces the distance between the original animal and vegetal poles, is the result of several factors. One contributing factor is the closure of the blastopore, which is narrowed in a posterior to anterior direction by cell intercalation at the posterior blastopore lip (zippering of the 3c^2^ and 3d^2^ progeny [29]). Here we describe a previously unrecognized process in which the anterior region (animal pole) is brought in closer proximity to the anterior edge of the blastopore by expansion of the provisional epithelium (e.g., 1q^2^ and 1d^121^,1d^122^ cells, Fig. 1d-f, and displacement of the 1q^1^ progeny). It is possible that proliferation of post-trochal cells also contributes to this process (Fig. 1a-c, such as those derived from the 2d clone, as proposed by [31, 32] or even the 3c and 3d clones may also drive this bending in *C. fornicata*).

The progeny of 4d do not likely contribute to these rearrangements. While removal of 4d (the embryonic organizer) early during development leads to radial development and prevents gastrulation, removal of 4d later during development (after its organizing role has ended) or ML and MR (which removes all progeny of 4d) does not affect blastopore formation, the development of the mouth, or inhibit elongation of the embryo (data not shown). On the other hand, it is possible that organizer signaling from 4d may play an indirect role, as it influences the behaviors of other cells in the embryo: possibly 1a^2^-1d^2^.

In the direct-developing nemertean *Carinoma tremaphoros,* the blastopore is also said to shift from the vegetal pole toward the ventral side of the embryo by expansion of 1q–derived cells [11]. But in this case, unlike in *C. fornicata*, the 1q–derived cells are highly proliferative and expand around the *posterior* pole [11]. Thus, it is likely that morphogenetic mechanisms involved in animal-vegetal axis bending, and in the displacement of the blastopore, may be highly variable between spiralian species. More careful examination in a wider range of embryos will be necessary to determine the full breadth of variation that might exist within this branch of bilaterians.

### Development of the prototroch

The embryonic origin of the primary trochoblasts from the 1q lineage is thought to be one of the more conserved features of spiralian development [38, 44]. Wilson [45] and Mead [46] where among the first to report that the 1q^2^ cells in particular contribute to prototroch cells in annelids, and referred to them as primary trochoblasts. Since then, additional classical descriptions, and a few modern lineage tracing studies, have confirmed that the primary trochoblasts come from the 1q^2^ cells and share other characteristics, such as cleavage arrest and cilia formation [47–49]. Therefore, it was surprising to discover that the 1q^2^ cells do not contribute to the prototroch in *C. fornicata*. Hence, in *C. fornicata* the prototroch is derived entirely from the 1a^1^-1c^1^ daughters (Figure 3), where the prototroch is mainly formed by 1b^1^ (ventro-laterally), and to a lesser extent by 1a^1^ (on the posterior-left side) and 1c^1^ (on the posterior-right side).

Conklin [40] mentioned that the 1q^2^ cells do not divide again until possibly very late during cleavage. He refers to these four cells as “turret cells,” which eventually lie between the arms of the molluscan cross. The “molluscan cross” represents a configuration of cells in which the 1q^1^ progeny make up the four radial arms of a cross, which intersects at the animal pole. The outer tips of those arms are made up of the animal-most progeny of the second quartet micromeres. One of the arms of this cross is labeled in Fig. 4g. Just as we report here, Conklin [40] states that “the 1q^2^ turret cells become the largest cells (save the vegetal macromeres) in the entire embryo.” His monograph only reports seeing a single division ever taking place in some 1a^2^ and 1b^2^ cells, which he also shows in a few illustrations. Likewise, except in one schematic diagram (his number 11), the 1c^2^ and 1d^2^ cells are never described to undergo further divisions. In fact, there is confusion as to whether any of these cells divide at all, as Conklin [40] goes on to state on page 106 and again on page 107, that “In *Crepidula* I have never seen these cells divide though likely the anterior ones (1a^2^ and 1b^2^) do divide at a later stage,” and “the posterior turret cells (1c^2^ and 1d^2^) divide very seldom, if at all.” Conklin speculates on page 59 that: “In *Crepidula* at least two of these cells (1a^2^ and 1b^2^), and probably all four, form a portion of the velum: but because I am not certain as to the destiny of the posterior two cells (1c^2^ and 1d^2^), I prefer to call the group for the present by a non-committal name (“turret cells”). Their destiny has not been determined in any other form.” Even so, Conklin [40] considered the turret cells to be homologous with trochoblast cells of annelids, and states (without any evidence) that the progeny of the anterior turret cells (1a^2^ and 1b^2^) give rise to the first row of prototroch cells, and that the posterior turret cells (1c^2^ and 1d^2^), together with large cells derived from the basal and middle cells of the posterior arm (derived from 1d^1^), give rise to the head vesicle. Here, we clearly show that those cells do not contribute to such structures.

Another function that has been attributed to the 1q cells is their role in specifying the D quadrant in equal-cleaving spiralians [50]. This is accomplished specifically by the 1q^1^ daughters. Recently, we have shown that the 1q^2^ cells do not play a role in the specification of the D quadrant in *C. fornicata* (in press).

It is likely of significance that the 1q^2^ cells in *C. fornicata* exhibit behaviors that are similar to the trochoblasts present in other species. Like those cells, *C. fornicata* 1q^2^ cells become cleavage-arrested after their birth, and they become highly ciliated (Fig. 5). We argue that in *C. fornicata*, there has been a developmental shift (in terms of both their division timing and ultimate placements, e.g., heterochronic and heterotopic shifts) in the developmental program of these 1q^2^ cells relative to that seen in other species. As a consequence, these cells are not incorporated into the prototrochal ciliary band and are ultimately lost from the larva. Another explanation for this novel behavior of the *C. fornicata* 1q^2^ cells could be that their unusual ability to stretch causes them to apoptose. Although we have not confirmed that the loss of the 1q^2^ and 1d^1^-derived cells is a result of the classic apoptosis pathway, we note that apoptosis has been linked to cell stretching in several models, for example in myoblasts [51].

In the direct-developing leech *Helobdella*, the primary quartet micromeres also contribute to both definitive tissues like neurons in the supraesophageal ganglion and longitudinal muscles, as well cells in the provisional integument which are lost from the embryo [52]. The specific contributions of the 1q^1^ versus 1q^2^ cells have been examined by modern lineage tracing in only a handful of other spiralian species. For example, in the limpet *Patella* [48, 53] the polychaete *Capitella* [49], and the nemertean *Carinoma* [11], the 1q^2^ cells do indeed contribute to the prototroch. In the gastropod *Ilyanassa*, the sub-lineages of the 1q cells have not been investigated specifically, but 1q labeled clones [30] show large cells on the dorsal side of the head that might indicate that the 1q^2^ lineage is similar to that described for *C. fornicata*. In a study using acetylated tubulin antibody staining to follow the development of *Ilyanassa* ciliary bands, Gharbiah et al. [54] showed large ciliated cells in the anterior of the early embryo. Some of these large cells could be 1q^2^ cells that may not contribute to the prototroch. The large acetylated tubulin-positive cells in the anterior-dorsal region of the early *C. fornicata* embryo come from the 1q^2^ cells, and to some extent the 1q^1^ progeny (i.e., 1d^1^ progeny). Careful lineage tracing of sub-lineages within the primary quartet in other spiralians will be useful in the future to reveal whether other species might have also undergone these evolutionary/developmental transitions.

## Methods

### Animal care and handling

Adult *Crepidula fornicata* were obtained from the Marine Resources Center at the Marine Biological Laboratory in Woods Hole, MA. Embryos were obtained and reared, as previously described [35, 41, 42]. Briefly, embryos were reared in gelatin-coated 35 mm petri dishes in a total volume of 3 ml to 4 ml FSW containing penicillin and streptomycin (see [41]).

### Lineage tracing

Each of the 1q^1^ and 1q^2^ cells were pressure micro-injected with either Rhodamine Green dextran (cat. no. D-7153, Life Technologies, Grand Island, NY) or DiIC18 (cat. no. D-282, Life Technologies, Grand Island, NY) at the 16- to 28-cell stages, as previously described [29, 37, 39, 41, 55]. To facilitate the injections, the nuclei were pre-labeled by expression of GFP tagged histone H2B protein via injection of synthetic RNA into zygotes (following [29]). These embryos were subsequently reared to various stages for clonal analyses of the injected cells’ progeny. At least twenty embryos were examined for each of the eight different 1q^1^/1q^2^ labeled cell clones.

### Fixation, histology and microscopy

Specimens were fixed for one hour in a 4% solution of formaldehyde (Ted Pella, Inc., Redding, CA) diluted in filtered sea water (FSW) for 1 h at room temperature. When prepared using a 16% formaldehyde stock solution the osmolarity of the fixative solution was adjusted using Instant Ocean Reef Crystals (Spectrum Brands, Blacksburg, VA) at a concentration of 0.38 g per 40 ml of working volume. Immediately after fixation, the formaldehyde solution was removed and replaced by three washes of 1X PBS (1X PBS:1.86 mM NaH_2_PO_4_, 8.41 mM Na_2_HPO_4_, 175 mM NaCl, pH 7.4) and three 100% methanol washes. Samples were stored in 100% methanol at −80 °C, until further use. Nuclei were labeled in a 0.5μg/ml solution of DAPI (Life Technologies, Grand Island, NY) in the dark for 10 min. To reduce background fluorescence, the DAPI solution was removed and samples were washed three times in 1X PBS/0.1% Tween. Specimens were stored in 80% glycerin/20% 1X PBS at 4 °C. Embryos were photographed live following gentle compression under a coverslip using Zeiss M2 Imager epi-fluorescence microscope.

### Immunohistochemistry

Antibody localization was performed using anti-acetylated tubulin (1:400, #T-7451, Sigma, St. Louis, MO) and follows the method described in Giani et al. [56]. Specifically, embryos were rehydrated in 1X PBS, permeabilized in 0.5% Triton X-100/1X PBS and blocked in 0.5% Triton X-100/1X PBS/10% normal goat serum for 2 h at room temperature. Anti-acetylated tubulin antibody was added at a concentration of 1:400 and incubated overnight at 4 **°**C. The next day, primary antibody was removed and embryos were washed four to five times in 1X PBS/0.5% Triton X-100 over the course of one hour. Secondary antibody (anti-mouse Alexafluor 546, 1:400) was added to the embryos and incubated at room temperature in the dark for 2 h (or alternatively overnight at 4 **°**C). Secondary antibody was removed and embryos were washed four to five times with 1X PBS/0.5% Triton over the course of one hour at room temperature. Subsequent incubation in DAPI (0.5μg/ml, Life Technologies, Grand Island, NY) dissolved in 1X PBS allowed visualization of nuclei. Embryos were incubated in this solution in the dark for 20 min, washed two to three times in 1X PBS over the course of 30 min and mounted on slides in 80% glycerin/20% 1X PBS.

## Conclusions

Our data reveal how the spiralian trochoblast lineage has evolved. Although in other spiralians, the 1q2 cells are often said to cleavage-arrest after 1 to 2 rounds of divisions and contribute to the primary ciliary band, the *C. fornicata* 1q2 cells instead are cleavage-arrested at their birth, and are ultimately lost from the embryo, never contributing to the definitive prototroch. To our knowledge, this is the first example of its kind. However, modifications to the prototroch lineages have been documented in other species. Often in those cases cells in the prototroch are more proliferative, not less (for example in the case of the polychaete *Capitella* [49] and the nemertean *Micrura* [57]). We also discovered that the 1q2 cells in *C. fornicata* undergo morphogenetic events to form an expansive, flattened provisional epithelium on the dorsal/anterior region of the embryo. We hypothesize that this epithelium may function to facilitate animal-vegetal axialbending. Careful lineage tracing of primary quartet sub-lineages in other spiralians will be valuable in the future to reveal additional ways in which the trochoblast lineage has been modified over evolution.
